# A Manual of Surgery, Founded upon the Principles and Practice Lately Taught by Sir Astley Cooper and Joseph Henry Green, Esq., F. R. S., Professor of Anatomy to the Royal Academy, Surgeon to, and Lecturer on Surgery, St. Thomas’s Hospital

**Published:** 1833

**Authors:** 


Art. VII.—*A Manual of Surgery, founded upon the principles and practice lately taught by Sir Astley Cooper and Joseph Henry Green, Esq., F. R. S., Professor of Anatomy to the Royal Academy, Surgeon to, and Lecturer on Surgery, St. Thomas's Hospital.* Edited by Thomas Castle, F. L. S. Boston: Munroe and Francis. New York: Charles L. Francis. 1833. 12mo. pp. 460.

The advantages of a judicious and comprehensive manuel, to which the student of medicine may refer, for the purpose of acquiring an outline of the principles of the science he is studying, or by which the practitioner may revive the recollection of former studies, and call up associations which business or time may have driven from his memory, will be acknowledged by all. Of the numerous manuals which have, at different times, been offered to the public, we know of none that has higher claims upon the profession than this. It contains, by far, a more faithful abstract of the opinions of Sir Astley Cooper than any of the ephemeral works that have been published under the sanction of his name, concisely and perspicuously expressed. The more important principles are printed in larger type than the explanations, qualifications, &c., which are necessary to elucidate. By this arrangement we think an important advantage is offered to the student, his attention being kept alive and his memory assisted by the connexion which is thus preserved between principles and details.

We subjoin a few extracts, not only as specimens of the work, but for the important principles they contain.

Wounds of the brain will often happen without producing any interruption to the operations of either the mind or body. But should the wound be accompanied by compression or concussion, then the particular symptoms which characterize those injuries will be present.

If the wound is a simple incision or laceration, it will prove quite harmless. Indeed, it frequently occurs that considerable portions of the brain are lost, and yet the mental and bodily functions continue unimpaired. Epileptic fits and hemiplegia certainly sometimes follow these injuries.

It occasionally happens, when a portion of brain has been lost, that a piece of the cranium will, by being driven in, occupy its place.

If in these cases, no symptoms of compression manifest themselves, you must not elevate the depressed bone; for were you to do so, you would, in all probability, give rise to extravasation, or increase the hazard of inflammation.

The danger attending injuries of the brain, arises principally from two causes; the formation of fungus, and from inflammation.

*Fungus:*—When the brain receives a wound, you must commence your curative exertions, by abstracting as large a quantity of blood from the system as the constitution of your patient will bear; not, however, to such an extent as to prevent the restorative operations of nature. After some days have elapsed, the divided parts begin to unite by means of the adhesive inflammation; but if this process cannot effect a cure, granulations form, which at length project through the opening in the skull, and give rise to fungus.

When this is the case, the safety of your patient will depend on the treatment you adopt; for if you do not repress the growth of the fungus until the dura mater heals over it, there will be violent constitutional irritation, and the life of the patient in jeopardy; but on the contrary, if you attend to the condition of the wound, and prevent the fungus from rising, there will be comparatively speaking, but little danger.

Your treatment should be as follows:—Apply to the fungus a piece of lint wetted with liquor calcis, and over this, strapping of adhesive plaster; when you examine the part on the following day, you will find the fungus considerably diminished: you are then to use a thicker piece of lint, and the strapping as before; you must pursue this plan until you have succeeded in getting it even with the dura mater, in which position it must be cautiously preserved; when, at last, the dura mater heals over it, and your object is accomplished.

*Inflammation:*—Wounds of the brain are dangerous from the inflammation which supervenes, and which danger is increased, if the dura mater is the part attacked. When inflammation attacks the membranes, symptoms of irritation will be present: but if the brain itself, they will be those of compression.

The ordinary symptoms are, great pain in the head; the scalp œdematous round the external wound; the edges of the wound have a shining, glossy appearance; and there is a discharge from the wound itself, composed of serum and blood; sometimes the parts about the wound have a sloughy appearance; the countenance is very much flushed, and the carotid arteries beat with very great force; there are very frequent rigors; sometimes hemiplegia; and the patient very quickly falls into a comatose state.

Inflammation of the brain is more slow in its occurrence after an injury, than inflammation after an injury of another part. It is generally about a week before it comes on; very rarely under that time.

It often happens that inflammation of the brain does not come on till a fortnight, or even three weeks after the injury; you must, therefore, be upon your guard, as your patient is not out of danger until that length of time has elapsed, and sometimes not even then.—p. 73–5.

The following remarks upon the application of the trephine.

When the dura mater is wounded in this operation, the patient very rarely recovers; but if the dura mater and pia mater are both injured, the danger is less: in the former case there will be excessive inflammation; whereas, in the latter, a fungus immediately projects and fills up the cavity.

If the whole depressed portion of bone cannot be raised, or all the blood or matter cannot be discharged, by making one perforation, the operation must be repeated, and as often as the case may require.

Removing large portions of the skull, however, should always be avoided if possible, because it may be itself the source of bad and even fatal consequences; but it is certainly less dangerous, than not completely removing the pressure from the surface of the brain.

The operation in question, we have observed, is not always necessary; but there are four circumstances when it becomes requisite.

First:—When there is extravasation of blood between the dura mater and skull.

Secondly:—In compound fracture with depression, *unaccompanied* with symptoms of compression.

Thirdly:—In simple fractures, with depression and symptoms of compression continuing after depletion.

And lastly:—It is sometimes required that an opening should be made in the cranium, when there is matter between the dura mater and skull.

Now it generally happens in this last case, where there is matter between the dura mater and skull, that there is fracture, and this is an indication that some injury has been done to the brain, when it is also followed by rigors and other symptoms: still it will be right, in some cases where there is no fracture, and the other symptoms, rigors, &c. are present, to penetrate the bone, to see whether matter is lodged between it and the dura mater; and this is the only case in which it is proper.—p. 77–8.

Wounded arteries require so much despatch and presence of mind upon the part of the surgeon that the proper mode of affording relief in such cases should always be before the mind of the practitioner.

*Arteries of the Scalp:*—Wounds of these arteries require a complete division of the injured vessel, and the application of pressure.

By the first, retraction is permitted, and future bleeding prevented; by the second, the present hemorrhage is suppressed.

*Axillary Artery:*—In taking up the axillary artery, when it is wounded, Scarpa recommends the opening, made to expose the vessel, should be sufficiently extensive; and that the vessel should be raised from plexus of the nerves before it is secured.

In performing this operation, agreeable to Scarpa's directions, an assistant must compress the vessel, from above the clavicle, as it passes over the first rib. When the weapon has penetrated from below upward directly into the axilla, the surgeon is to make a free dilatation of the wound upon a director, or his finger. This must be done to a sufficient height to expose a considerable portion of the artery, and the precise situation of the wound in it.

When the weapon has pierced obliquely, or from above downwards, through a portion of the great pectoral muscle, into the axilla, the surgeon is to cut through the lower edge of this muscle, and enlarge the wound, on a directory, or his finger, so as to bring fairly into view the injured part of the artery. The thoracic arteries divided in the operation, must be immediately tied. The clots of blood are then to be removed, and the bottom of the wound cleaned with a sponge, by which means the opening in the axillary artery will be more clearly seen. Two ligatures will be required; one above the wound of the artery, and the other below.

*Brachial Artery:*—In tying the brachial artery, there is only one circumstance to bear in mind, and it is this; the vessel is accompanied by the medium nerve: now, if you should include this in the ligature, it would either destroy the patient's life, or cause paralysis of the limb.

Sometimes, in wounds of the brachial artery, the injury may be overcome by a slight bandage, and a thick dossil of lint as a compress. But if aneurism forms, the torniquet should be employed, and if this does not succeed, a ligature must be applied. The direction for the incision is the inner edge of the biceps muscle, and this cut almost immediately lays bare the median nerve.

*Ulnar Artery:*—The wounds of this artery are usually at the lower part of the forearm, where the vessel is situated, between the flexor carpi ulnaris and the flexor profundis: it is accompanied by the ulnar nerve, which must be carefully excluded from the ligatures.

When this artery is required to be secured, what is the anatomical direction for the incision? Why the tendon of the flexor carpi ulnaris: if you make your cut upon the inner side of this tendon, you will directly perceive the ulnar artery and ulnar nerve. This then is the part where the vessel may be most easily and safely tied. Two ligatures will be required, one above, and the other below, to prevent hemorrhage from the free anastomosis with the radial artery.

*Radial Artery:*—This artery is much more frequently wounded than the ulnar, being, in every respect, more exposed.

In securing the radial artery, let your incision be made on the radial side of the tendon of the flexor carpi radialis, and you will immediately find the artery close to its edge. The application of two ligatures is also necessary. Instead of putting ligatures on these vessels at the wrist, for aneurism, or wounds of the palmar arch, it has been recommended to employ pressure, by means of cork folded in lint, and bound down by a bandage. This practice when used, leads to great inflammation and irritation, and I would advise you against using pressure generally, and more especially as regards the ulnar and radial arteries, as they can be so readily tied.

*Anterior Tibial Artery:*—This vessel is rarely wounded at the upper part of the limb, but frequently at the lower and middle portions; consequently, it requires to be secured at different parts of its course.

The anterior tibial artery passes forward between the bones of the leg, about an inch below the upper head of the fibula.

In order to take up the vessel in this situation, a free cut must be made through the fascia, extending between the heads of the tibia and fibula. The incision is then to be continued more deeply at the edge of the peronæus longus, following the fascia between this muscle and the origin of the extensor digitorum communis. The artery will be met with on the interosseous ligament.

Another situation, in which this artery is sometimes required to be secured, is a little above the middle of the leg.

The finger is to be passed along the outer side of the spine of the tibia, and the breadth of the tibialis anticus muscle is to be ascertained. Along the outer margin of this muscle, an incision is to be made through the integuments and fascia, two inches and a half in length. The knife is then to be introduced between the outer margin of the tibialis anticus muscle, and the extensor longus of the great toe. In this space, at the depth of about an inch, the anterior tibial artery is situated.—p. 149–152.

We subjoin also Sir Astley's mode of puncturing the bladder through the perinuæm for suppression of urine:

*Operation through the Perinæum:*—This operation bears a great similitude to that for stone. It consists in puncturing the bladder by the side of the prostate gland, and is unattended with any danger of wounding the peritoneum, because it does not reach the anterior part of the bladder.

You make an incision in the perinæum, as in operation for stone, cutting down on the bulb of the penis, and drawing it to the right side. You then carry the knife within the ramus of the ischium till it reaches the prostate gland, which you push to the patient's right side. You then carry the instrument *obliquely upwards* into the bladder, your finger resting on the prostate gland.—p. 208–9.

Sir A., however, thinks that this operation is in general perfectly unnecessary.

We have now gone through the three different operations for puncturing the bladder, and although there is no great difficulty in either, I affirm that we ought to avoid all these operations, and that puncturing the bladder for stricture in the urethra, or enlargement of the prostate gland, is in general perfectly unnecessary.

In cases of accumulation of urine in the bladder, for which many would have recourse to puncturing that organ, I have found opening the urethra only, a better and safer course. All I do is this, I desire the patient to draw up his legs as if he was going to be operated on for stone; I then make my incision into the urethra, according to the seat of the stricture, and the result is, that the urine is passed by the perineum, and the bladder is relieved, without being in the slightest degree injured.—p. 209.

